# Supplementary material for: Interactions between Kazachstania humilis Yeast Species and Lactic Acid Bacteria in Sourdough
Source: Microorganisms. 2020 Feb 11;8(2):240. doi: 10.3390/microorganisms8020240 (PMC7074792; doi:10.3390/microorganisms8020240)
Supplement: Supplementary file 1 [file microorganisms-08-00240-s001.zip › microorganisms-681294-supplementary-final/FigureS2.pdf]

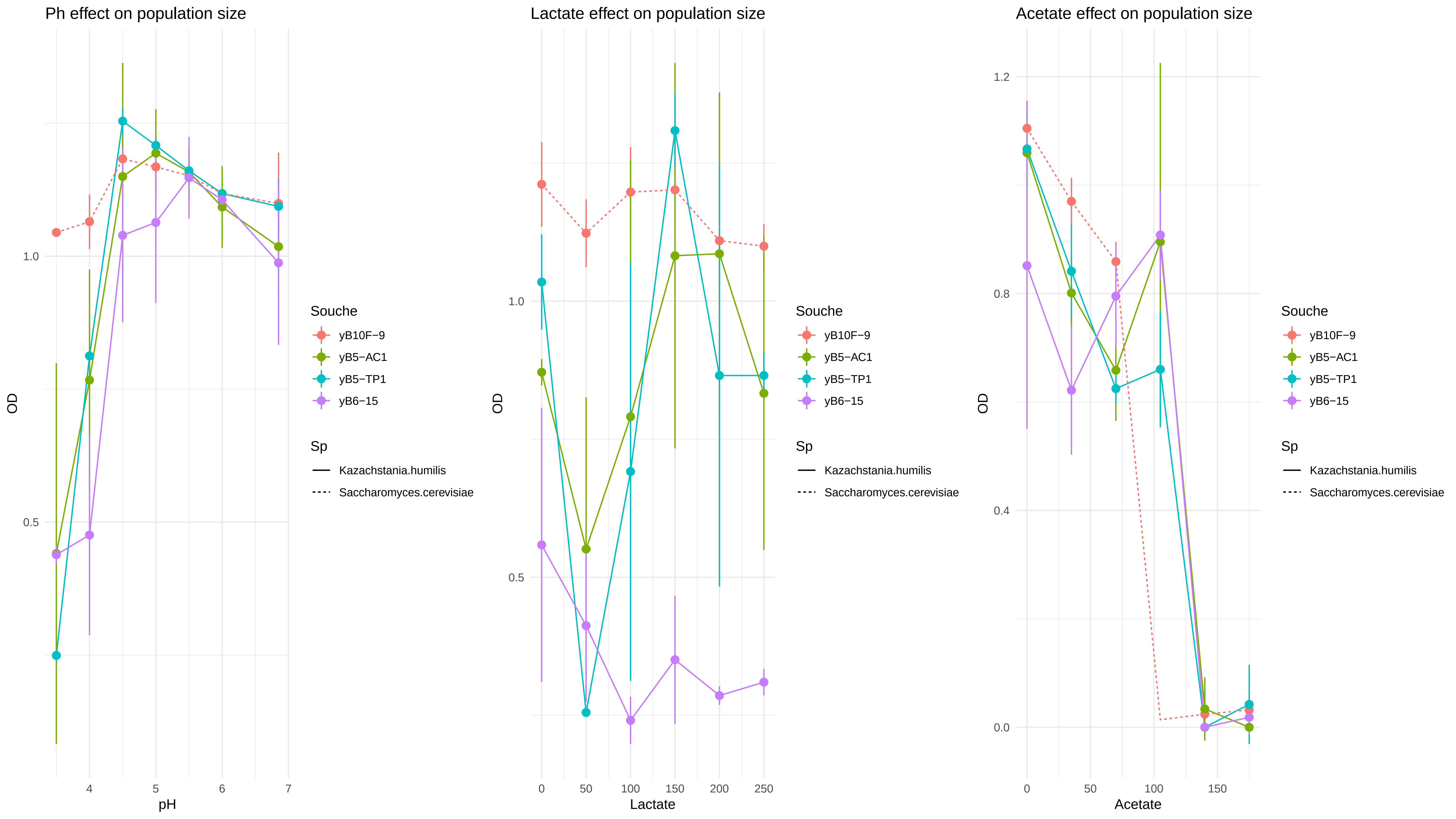

Ph effect on population size
Lactate effect on population size
Acetate effect on population size
OD
pH
Lactate
Acetate
Souche
yB10F−9
yB5−AC1
yB5−TP1
yB6−15
Sp
Kazachstania.humilis
Saccharomyces.cerevisiae
