# Supplementary material for: Interactions between Kazachstania humilis Yeast Species and Lactic Acid Bacteria in Sourdough
Source: Microorganisms. 2020 Feb 11;8(2):240. doi: 10.3390/microorganisms8020240 (PMC7074792; doi:10.3390/microorganisms8020240)
Supplement: Supplementary file 1 [file microorganisms-08-00240-s001.zip › microorganisms-681294-supplementary-final/SupplementaryTables.pdf]

Table S1. Bacteria strains assimilation of different substrates as analysed by API 50C (Biomérieux) .

**Bacteria**					D-Galactose	D-Raffinose	D-Saccharose	D-Glucose	D-Thréhalose	D-Maltose	Glycerol	Methyl-alpha	cycloheximid	acide lactique
Lactobacilus curvatus	bB16-cur	MTF 4123	B16	facultatively	+	-	+	+	+	+	-	NA	NA	NA
Lactobacilus sakei	bB4-sak	MTF 4118	B4	facultatively	+	-	+	+	+	-	-	NA	NA	NA
Lactobacilus sanfranciscensis	bB4-sf	MTF 3946	B4	obligately he	+	-	-	+	-	+	-	NA	NA	NA
Lactobacilus sanfranciscensis	bB5-sf	MTF 3945	B5	obligately he	-	-	-	+	-	+	-	NA	NA	NA
Lactobacilus hammesii	bB5-ham	MTF 3944	B5	obligately he	+	-	-	+	-	+	-	NA	NA	NA
Lactobacilus pentosus	bB5-pen	MTF 4114	B5	facultatively	-	-	+	+	+	+	-	NA	NA	NA
Lactobacilus kimchi	bB5-kim1	MTF 4117	B5	facultatively	+	-	+	+	+	+	-	NA	NA	NA
Lactobacilus kimchi	bB5-kim6	MTF 4116	B5	facultatively	+	-	+	+	+	+	-	NA	NA	NA
**Bacteria**					D-Fructose	D-Mannose	D-Mannitol	D-Arabinose	L-Arabinose	D-ribose	D-+ylose	D-Adonitol	Méthyl-betal	L-Rhamnose
Lactobacilus curvatus	bB16-cur	MTF 4123	B16	facultatively	+	+	-	-		+	-	-	-	-
Lactobacilus sakei	bB4-sak	MTF 4118	B4	facultatively	+	+	-		+	+	-	-	-	+
Lactobacilus sanfranciscensis	bB4-sf	MTF 3946	B4	obligately he	+	+	+	+	+	+	-	-	+	-
Lactobacilus sanfranciscensis	bB5-sf	MTF 3945	B5	obligately he	-	-	-	-	-	-	-	-	-	-
Lactobacilus hammesii	bB5-ham	MTF 3944	B5	obligately he	+	+	-	-	+	+	+	-	+	-
Lactobacilus pentosus	bB5-pen	MTF 4114	B5	facultatively	+	+	+	-	-	+	+	+	-	-
Lactobacilus kimchi	bB5-kim1	MTF 4117	B5	facultatively	+	+	-	-	+	+	+	-	+	-
Lactobacilus kimchi	bB5-kim6	MTF 4116	B5	facultatively	+	+	-	-	-	+	-	-	-	-
**Bacteria**					D-Sorbitol	Méthyl-alpha	N-AcéthylGlu	Amygdaline	Arbutine	Esculine	Salicine	D-cellobiose	D-Mélibiose	D-Mélézitose
Lactobacilus curvatus	bB16-cur	MTF 4123	B16	facultatively	-	-	+	-	-	-	+	-	-	-
Lactobacilus sakei	bB4-sak	MTF 4118	B4	facultatively	-	-	+	-	-	+	+	+	+	-
Lactobacilus sanfranciscensis	bB4-sf	MTF 3946	B4	obligately he	-	-	+	-	-	-	-	-	-	-
Lactobacilus sanfranciscensis	bB5-sf	MTF 3945	B5	obligately he	-	+	-	-	-	-	-	-	-	-
Lactobacilus hammesii	bB5-ham	MTF 3944	B5	obligately he	-	-	+	-	-	-	-	-	-	-
Lactobacilus pentosus	bB5-pen	MTF 4114	B5	facultatively	+	-	+	-	+	+	+	+	-	-
Lactobacilus kimchi	bB5-kim1	MTF 4117	B5	facultatively	-	-	+	+	+	+	+	+	-	+
Lactobacilus kimchi	bB5-kim6	MTF 4116	B5	facultatively	-	-	+	+	+	+	+	+	-	+
**Bacteria**					Amidon	Gentiobiose	D-Ly+ose	D-Arabitol	Potassium Gluconate					
Lactobacilus curvatus	bB16-cur	MTF 4123	B16	facultatively	-	-	-	-	-					
Lactobacilus sakei	bB4-sak	MTF 4118	B4	facultatively	-	+	-	-	+					
Lactobacilus sanfranciscensis	bB4-sf	MTF 3946	B4	obligately he	-	-	+	+	+					
Lactobacilus sanfranciscensis	bB5-sf	MTF 3945	B5	obligately he	-	-	-	-	-					
Lactobacilus hammesii	bB5-ham	MTF 3944	B5	obligately he	-	-	+	+	+					
Lactobacilus pentosus	bB5-pen	MTF 4114	B5	facultatively	+	+	-	-	+					
Lactobacilus kimchi	bB5-kim1	MTF 4117	B5	facultatively	-	+	-	+	+					
Lactobacilus kimchi	bB5-kim6	MTF 4116	B5	facultatively	-	+	-	-	-					

Table S2. Yeast strains assimilation of different substrates as analysed by API ID32 C (Biomérieux) . Only sybstrates that are assimilated by at least on strain are shown.

**Species**	**Strain's code**	**Lab code**	**Baker**	**Main physio**	**Assimilated substrates**									
**Yeast**					D-Galactose	D-Raffinose	D-Saccharose	D-Glucose	D-Trehalose	D-Maltose	Glycerol	Methyl-alpha	cycloheximid	acide lactique
Saccharomyces cerevisiae	yB10F-9	MTF3947	B10	maltose-posi	+	+	+	+	-	+	-	+	-	+
Kazachstania humilis	yB6-15	MTF3948	B6	maltose-nega	+	+	+	+	+	-	+	-	+	-
Kazachstania humilis	yB5-TP1	MTF3949	B5	maltose-nega	+	+	+	+	+	-	-	-	-	-
Kazachstania humilis	yB5-AC1	MTF4070	B5	maltose-nega	+	+	+	+	+	-	-	-	-	-

Table S3. Analysis of variance of yeast CFUs after 24h.

	Df	Sum Sq	Mean Sq	F value	P-value
Yeast strain	3	17863	5954	7.38	0.003**
Residuals	14	11293	807		

Table S4. Analysis of variance of Lactic Acid Bacteria CFUs after 24h.

	Df	Sum Sq	Mean Sq	F value	P-value
LAB strain	7	11770838	1681548	10.2	0.00002***
Residuals	20	3301333	165067		

Table S5. Analysis of variance of dough height after 6h. Type corresponds to either bacteria or yeast.

	Df	Sum Sq	Mean Sq	F value	P-value
Block	1	1.8	1.8	29.74	1.77 E-05 ***
Type	1	10.125	10.125	167.26	9.31 E-12 ***
Strain	5	6.185	1.237	20.43	1.33 E-07 ***
Residuals	22	1.332			

Table S6. Analysis of variance of metabolite concentrations in monoculture.

Variable	Df	Sum Sq	Mean Sq	F value	P-value
Maltose					
Block	1	20.50	20.50	71.10	4.84 E-09 ***
LAB/Yeast/no	2	0.16	0.08	0.28	0.76

Strains (Type)	5	22.80	4.55	15.80	2.74 E-07 ***
Residuals	27	7.79	0.29	NA	NA
Glucose					
Block	1	4.70	4.70	74.30	3.10 E-09 ***
LAB/Yeast/no	2	3.48	1.74	27.50	3.07 E-07 ***
Strains (Type)	5	3.12	0.62	9.85	1.91 E-05 ***
Residuals	27	1.71	0.06	NA	NA
Fructose					
Block	1	0.05	0.05	4.85	0.04 *
LAB/Yeast/no	2	0.61	0.30	28.70	2.09 E-07 ***
Strains (Type)	5	0.17	0.03	3.11	0.02 *
Residuals	27	0.29	0.01	NA	NA
Glycerol					
Block	1	0.02	0.02	28.90	1.26 E-05 ***
LAB/Yeast/no	2	0.16	0.08	117.00	1.05 E-13 ***
Strains (Type)	5	0.09	0.02	25.10	3.43 E-09 ***
Residuals	26	0.02	0.00	NA	NA
Ethanol					
Block	1	0.44	0.44	18.20	2.32 E-04 ***
LAB/Yeast/no	2	11.70	5.85	244.00	1.41 E-17 ***
Strains (Type)	5	5.77	1.15	48.20	2.49 E-12 ***
Residuals	26	0.62	0.02	NA	NA
Pyruvate					
Block	1	3.83 E-06	3.83 E-06	14.8	6.93 E-04 ***
LAB/Yeast/no	2	1.07 E-06	5.37 E-07	2.08	0.15
Strains (Type)	5	1.40 E-06	2.81 E-07	1.09	0.39
Residuals	26	6.72 E-06	2.58 E-07	NA	NA
Acetate					
Block	1	0.04	0.04	226	2.48 E-14 **
LAB/Yeast/no	2	0.11	0.05	301	1.05 E-18 ***
Strains (Type)	5	4.75E-03	9.49E-04	5.29	1.76 E-03 **
Residuals	26	4.67E-03	1.80E-04	NA	NA
Lactate					
Block	1	1.48	1.48	277	2.18 E-15 ***
LAB/Yeast/no	2	4.38	2.19	411	2.14 E-20 ***
Strains (Type)	5	0.08	0.02	3.14	0.02
Residuals	26	0.14	0.01	NA	NA

Table S7. Analysis of variance of the interaction effect (CFUs) for each yeast strains. The interaction effect was tested by comparing the yeast's CFUs after 24h in monoculture and co-cultures with LAB strain.

Yeast strain	Df	Sum Sq	Mean Sq	F value	P-value
yB5-TP1					
Mono/Cocultures	4	142905	35726	112	8 E-31 ***
Residuals	75	23947	319		
yB6-15					
Mono/Cocultures	6	106532	17755	32	6 E-23 ***
Residuals	121	66659	551		
yB10F-9					
Mono/Cocultures	3	125696	41898	54	8 E-15 ***
Residuals	44	33993	773		
yB5-AC1					
Mono/Cocultures	3	36525	12175	30	7 E-12***
Residuals	60	24712	412		

Table S8. Analysis of variance comparing the LAB/yeast competition effect between yeast strains. The competition effect was measured as the ratio between the CFUs in co-culture over the CFUs in monoculture

	Df	Sum Sq	Mean Sq	F value	P-value
Yeast strains	3	0.048	0.017	0.28	0.83
Residuals	16	0.88	0.055		

Table S9. Analysis of variance of the interaction effect (CFUs) for each LAB strain. The interaction effect was tested by comparing the LAB's CFUs after 24h in monoculture and co-cultures with yeast strain.

	df	sumsq	meansq	statistic	p.value
bB16-cur					
yeast	1	21376	21376	0.2	0.65
Residuals	26	2663671	102449		
bB5-sf					
yeast	4	1932259	483065	2	0.16
Residuals	81	23457037	289593		
bB5-pen					
yeast	2	172933	86466	2	0.12
Residuals	41	1621739	39555		
bB5-ham					
yeast	3	2945287	981762	5	2.65 E-03 **
Residuals	76	14440092	190001		
bB5-kim1					
yeast	1	672145	672145	15	6.66 E-04 ***
Residuals	26	1170650	45025		
bB5-kim6					
yeast	1	1247616	1247616	20	2.06 E-04 ***
Residuals	22	1392744	63307		
bB4-sf					
yeast	3	1380913	460304	3	0.0392 *
Residuals	56	8660857	154658		
bB4-sak					
yeast	1	74817	74817	1	0.26
Residuals	22	1247423	56701		
