# Supplementary figures and images for: Interactions between Kazachstania humilis Yeast Species and Lactic Acid Bacteria in Sourdough
Source: Microorganisms. 2020 Feb 11;8(2):240. doi: 10.3390/microorganisms8020240 (PMC7074792; doi:10.3390/microorganisms8020240)

LAB strains

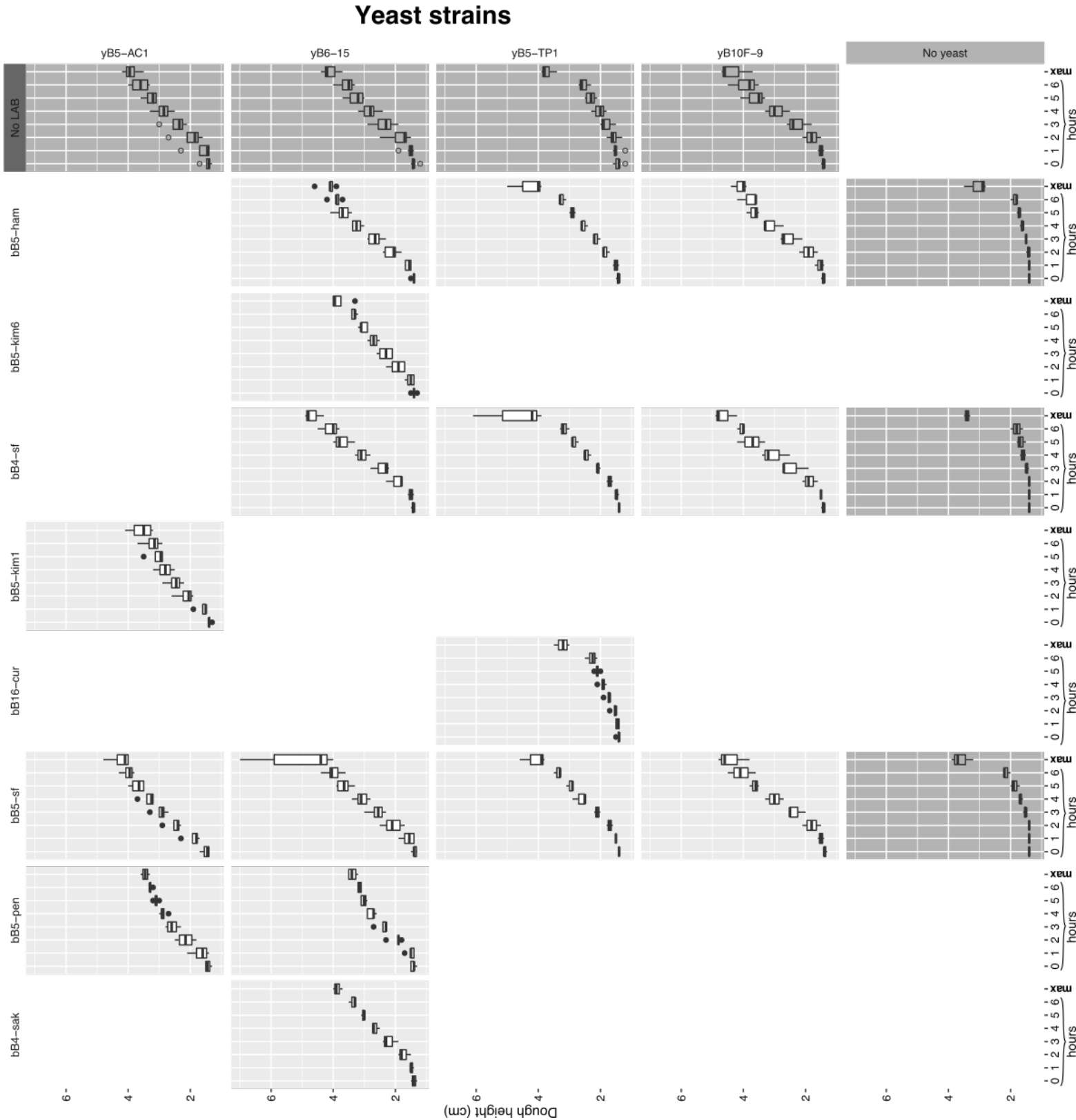

Supplement: Supplementary file 1 [file microorganisms-08-00240-s001.zip › microorganisms-681294-supplementary-final/FigureS1.pdf]

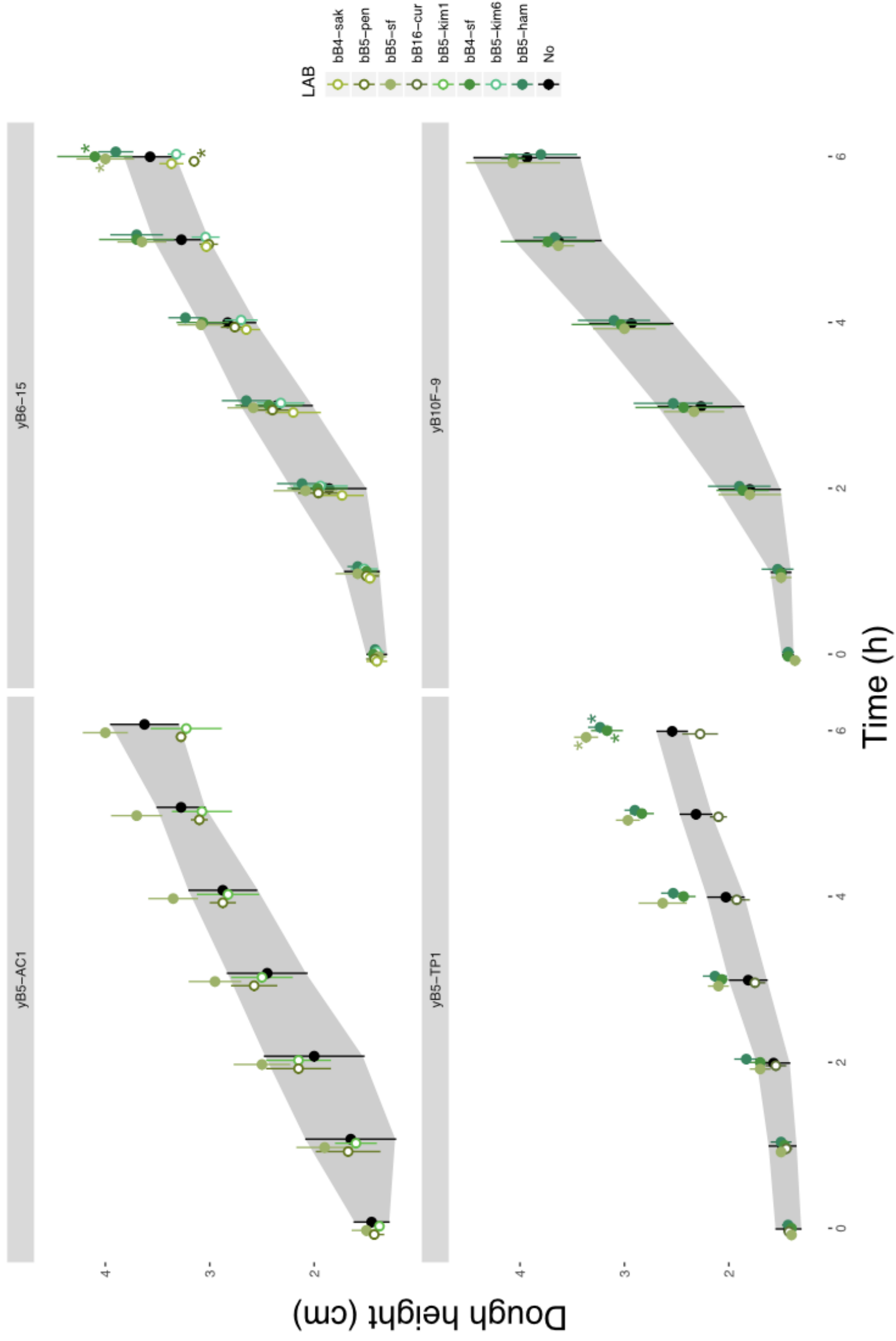

Supplement: Supplementary file 1 [file microorganisms-08-00240-s001.zip › microorganisms-681294-supplementary-final/figureS3.pdf]

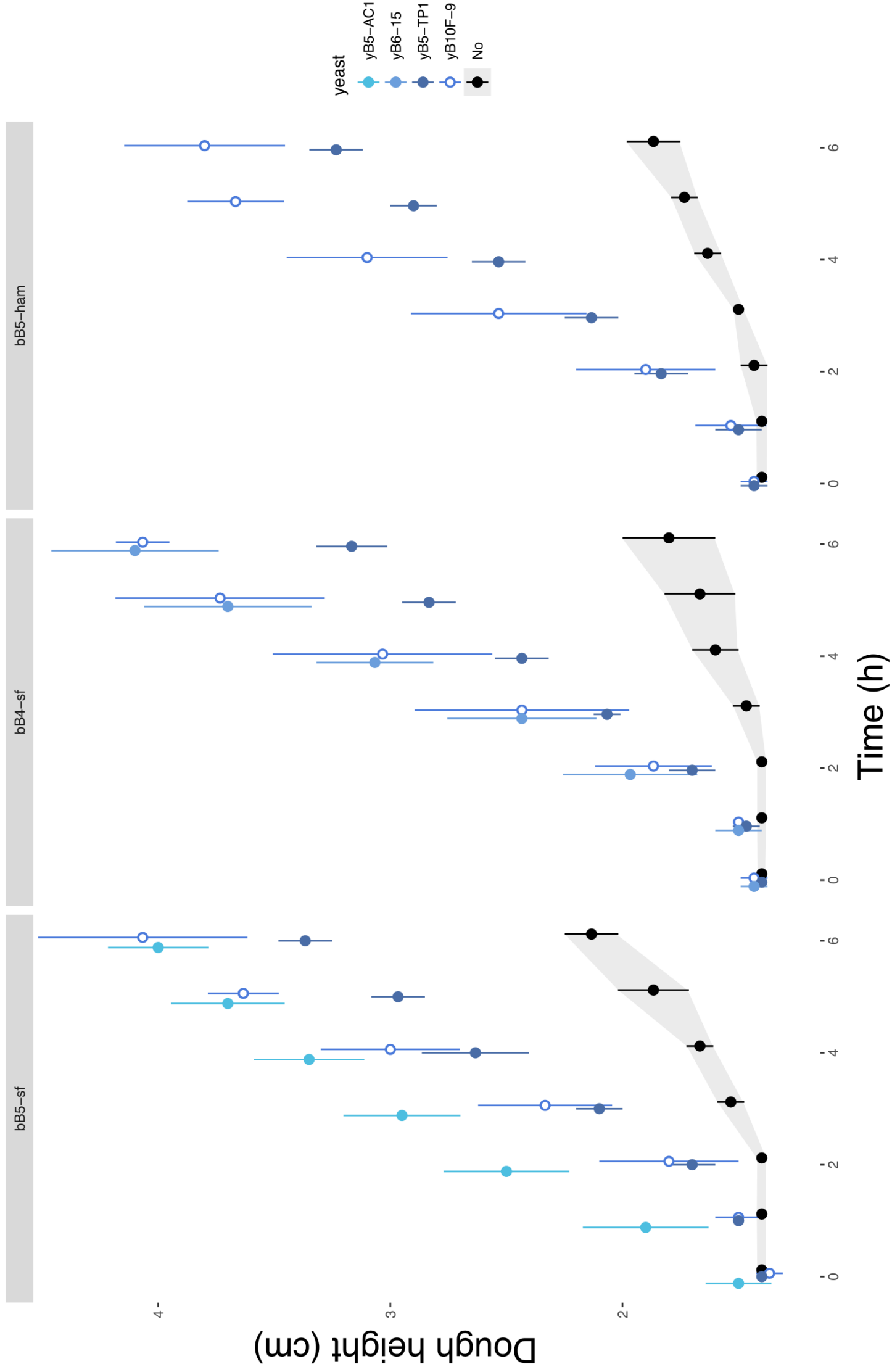

Supplement: Supplementary file 1 [file microorganisms-08-00240-s001.zip › microorganisms-681294-supplementary-final/figureS4.pdf]

glucose

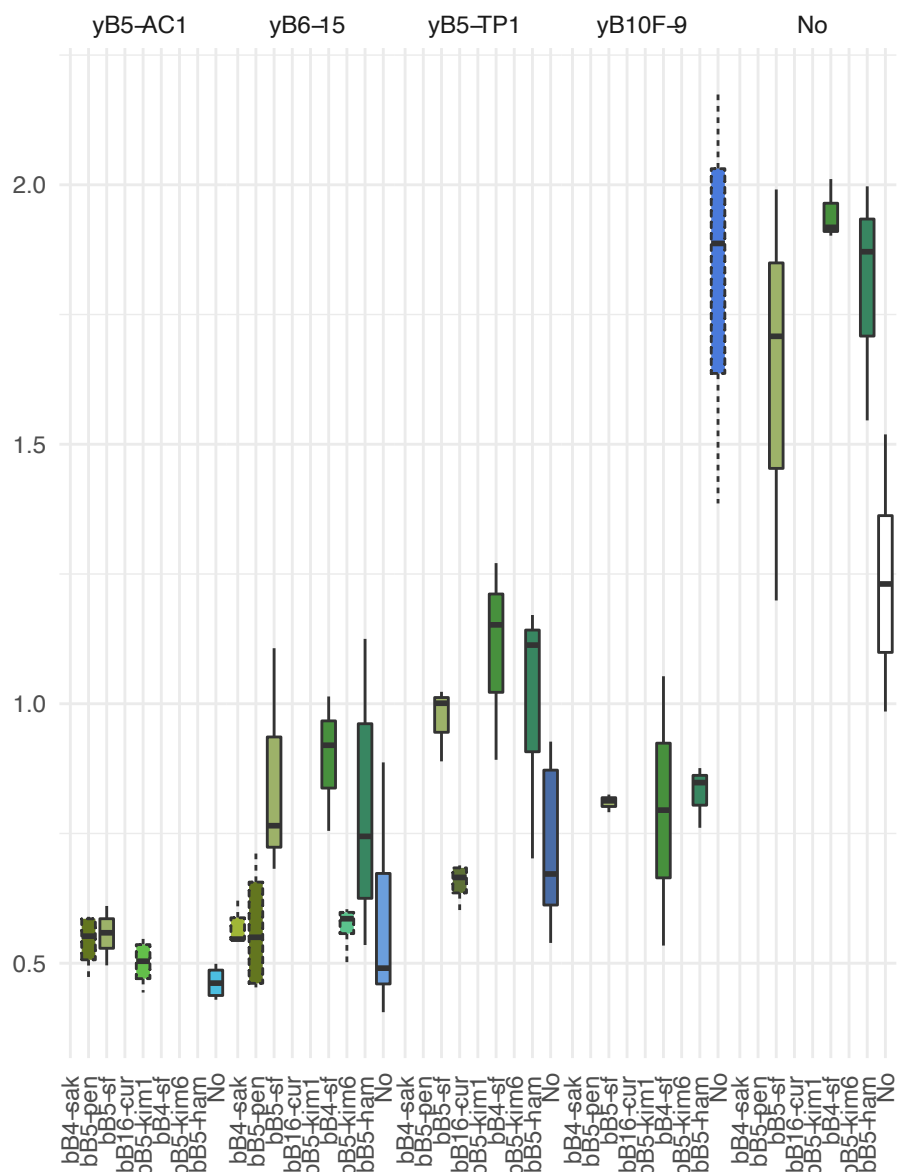

maltose

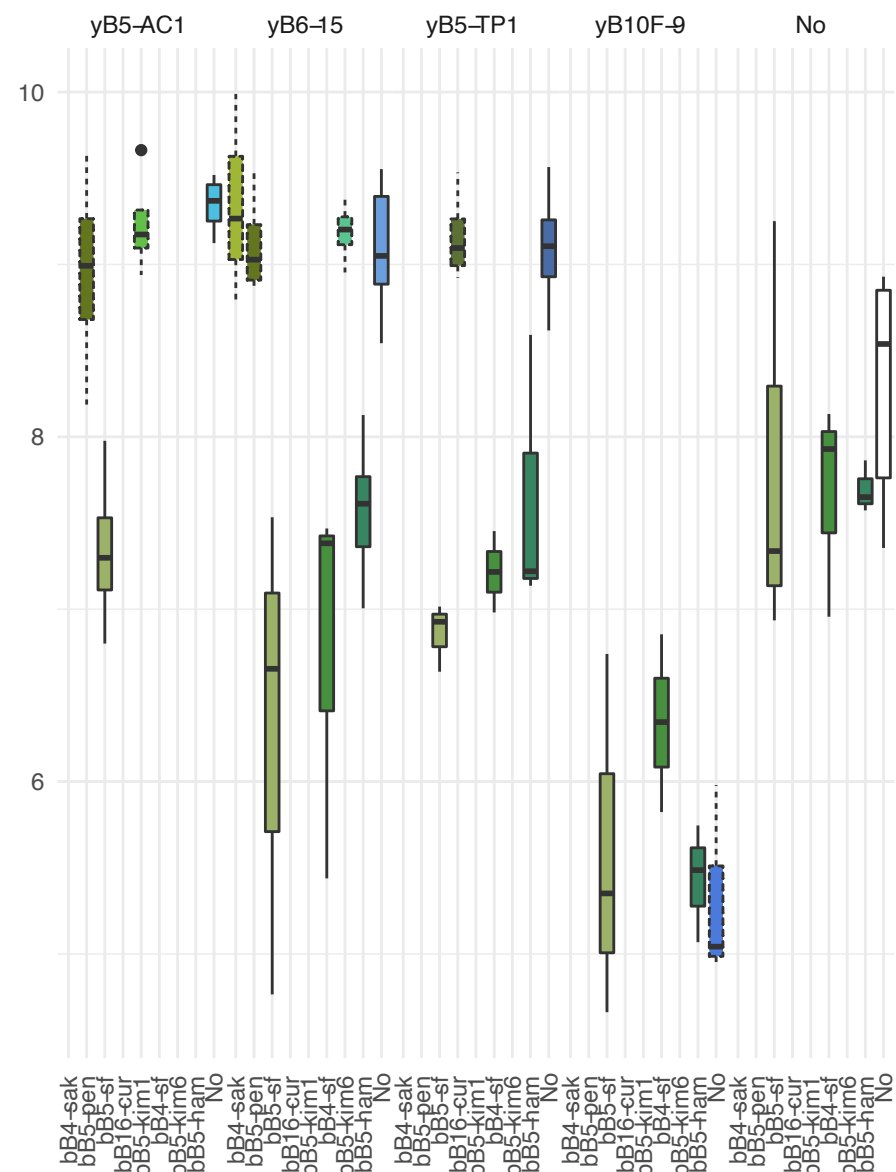

## fructose

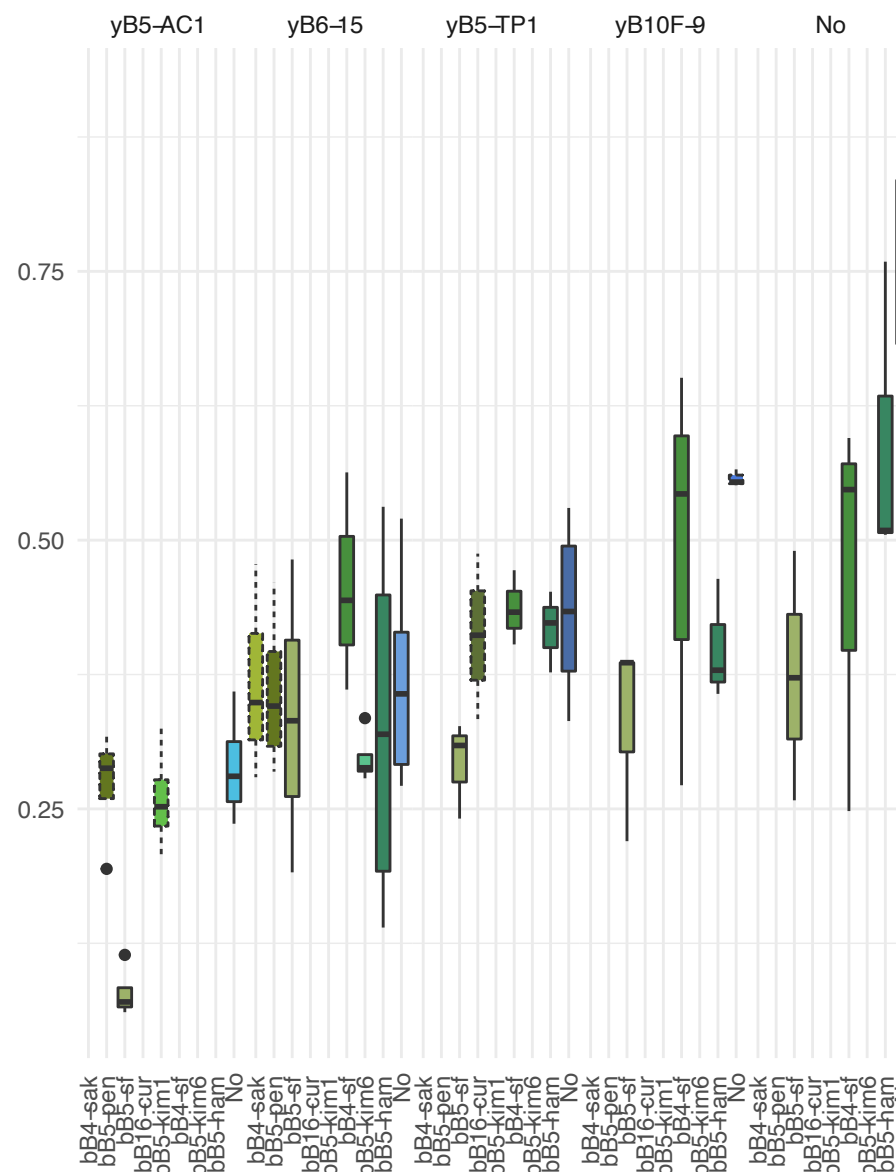

glycerol

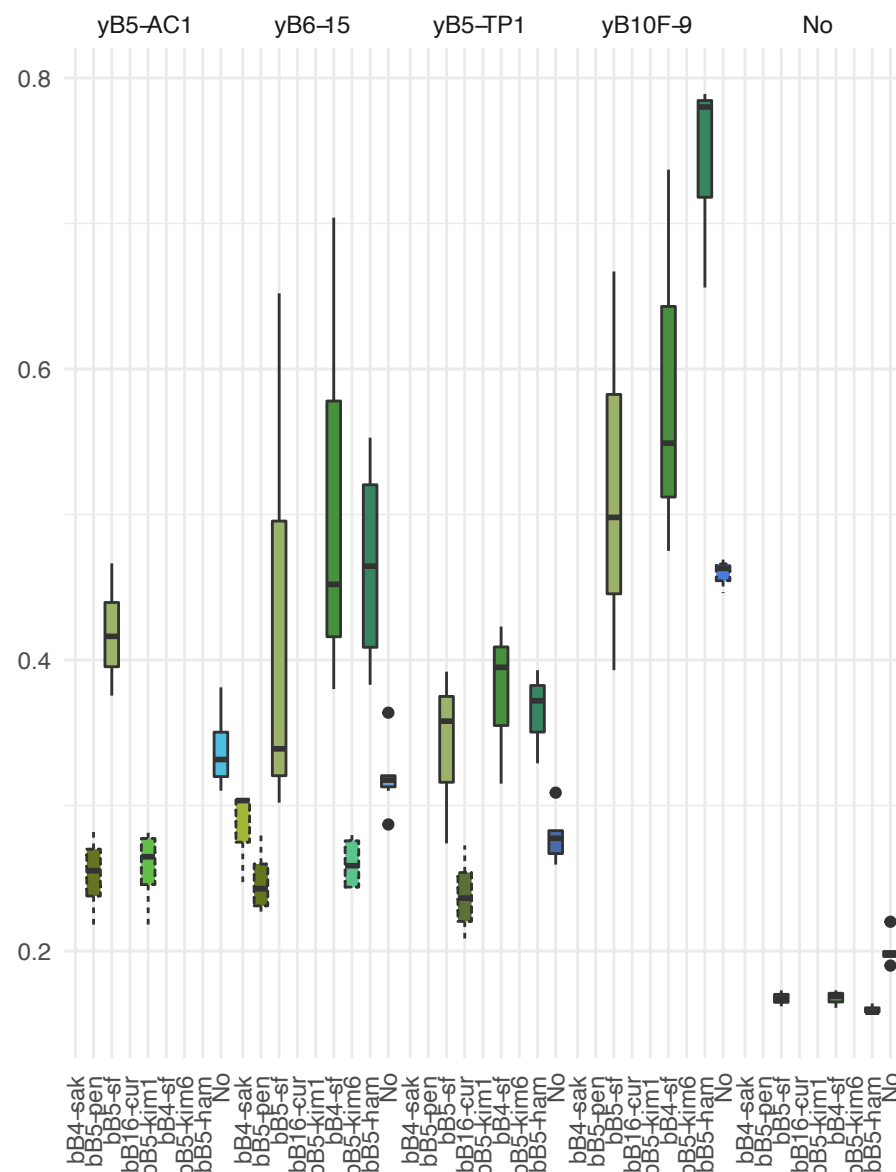

## ethanol

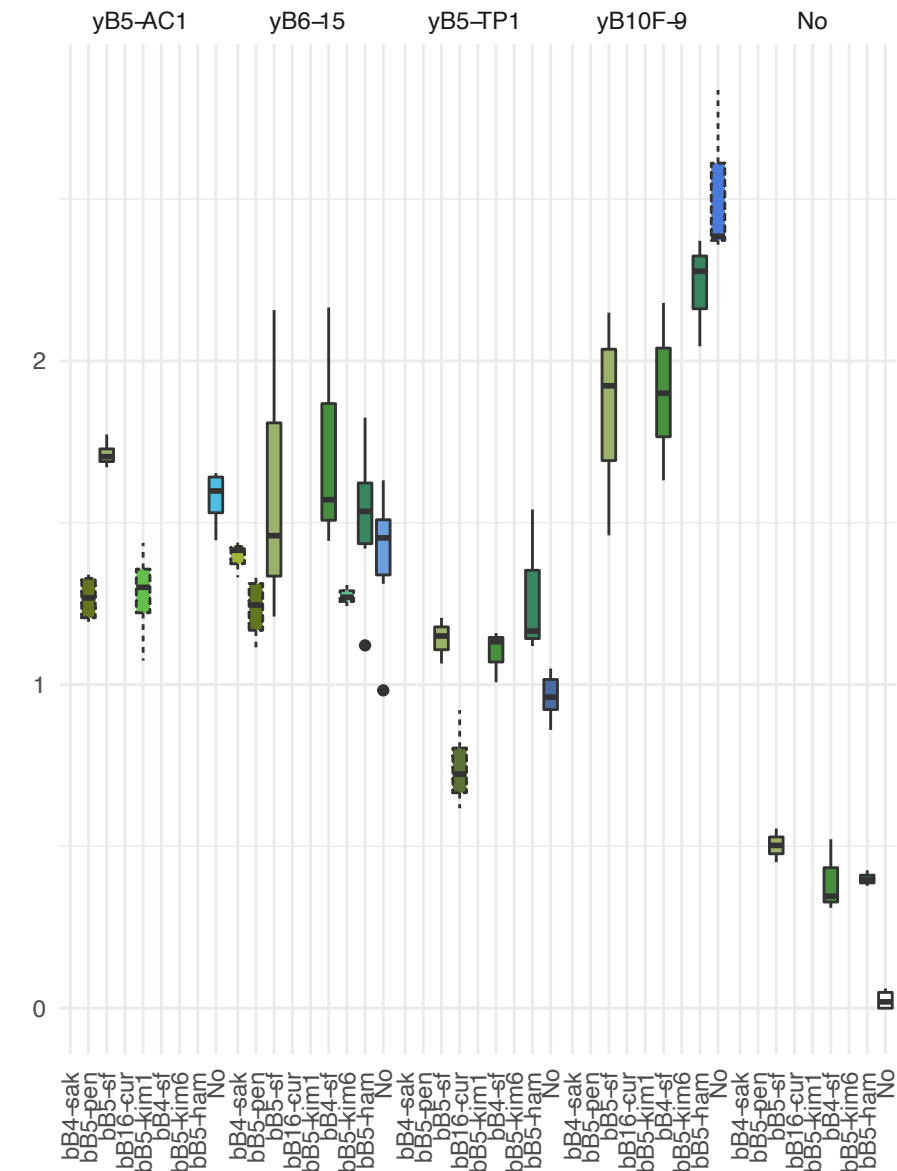

## pyruvate

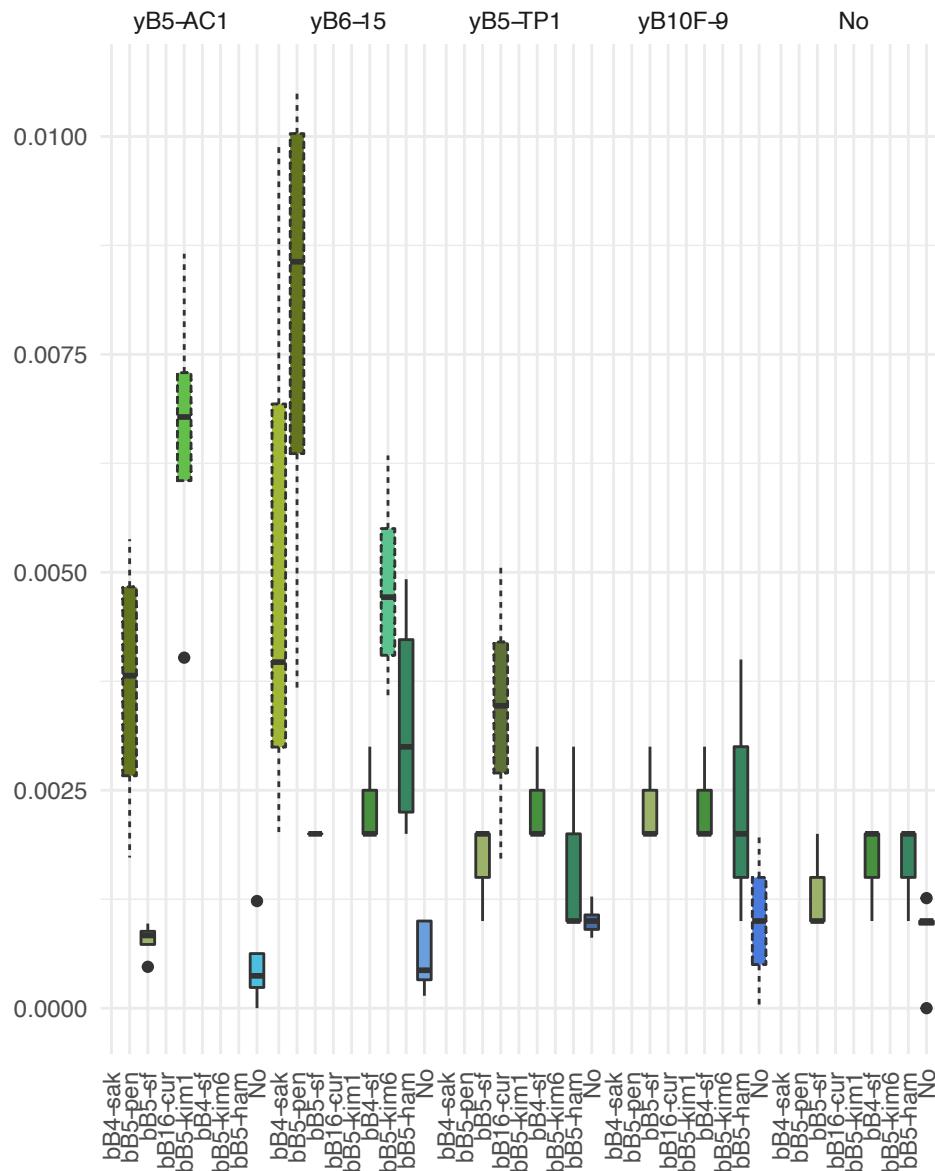

## acetate

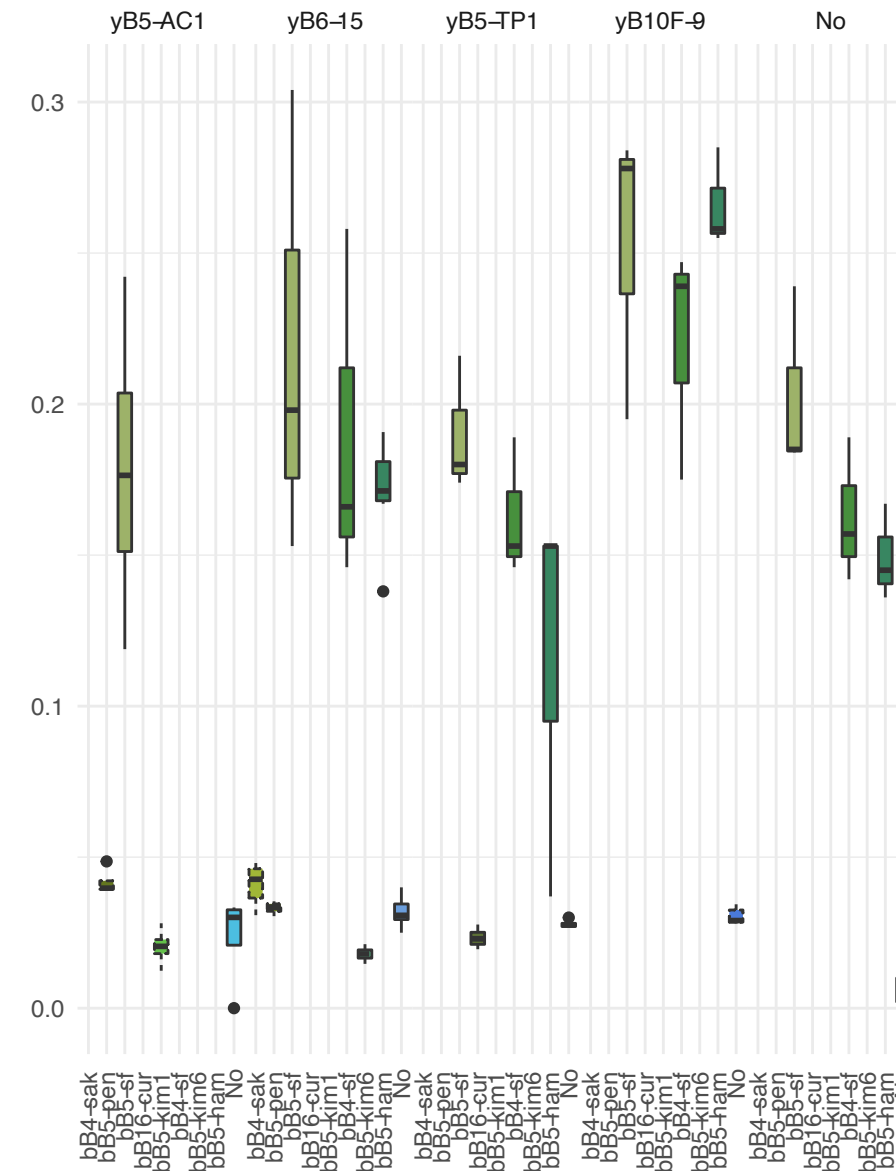

lactate

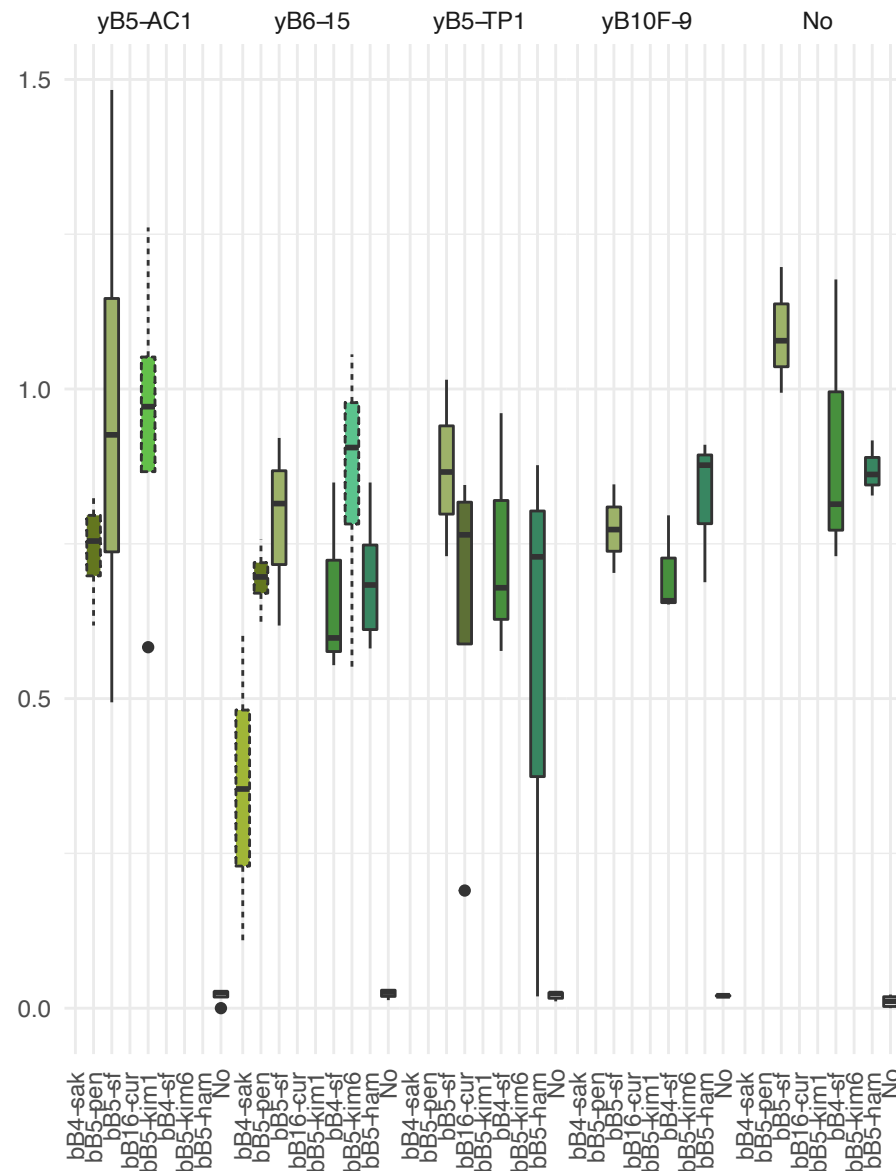

## succinate

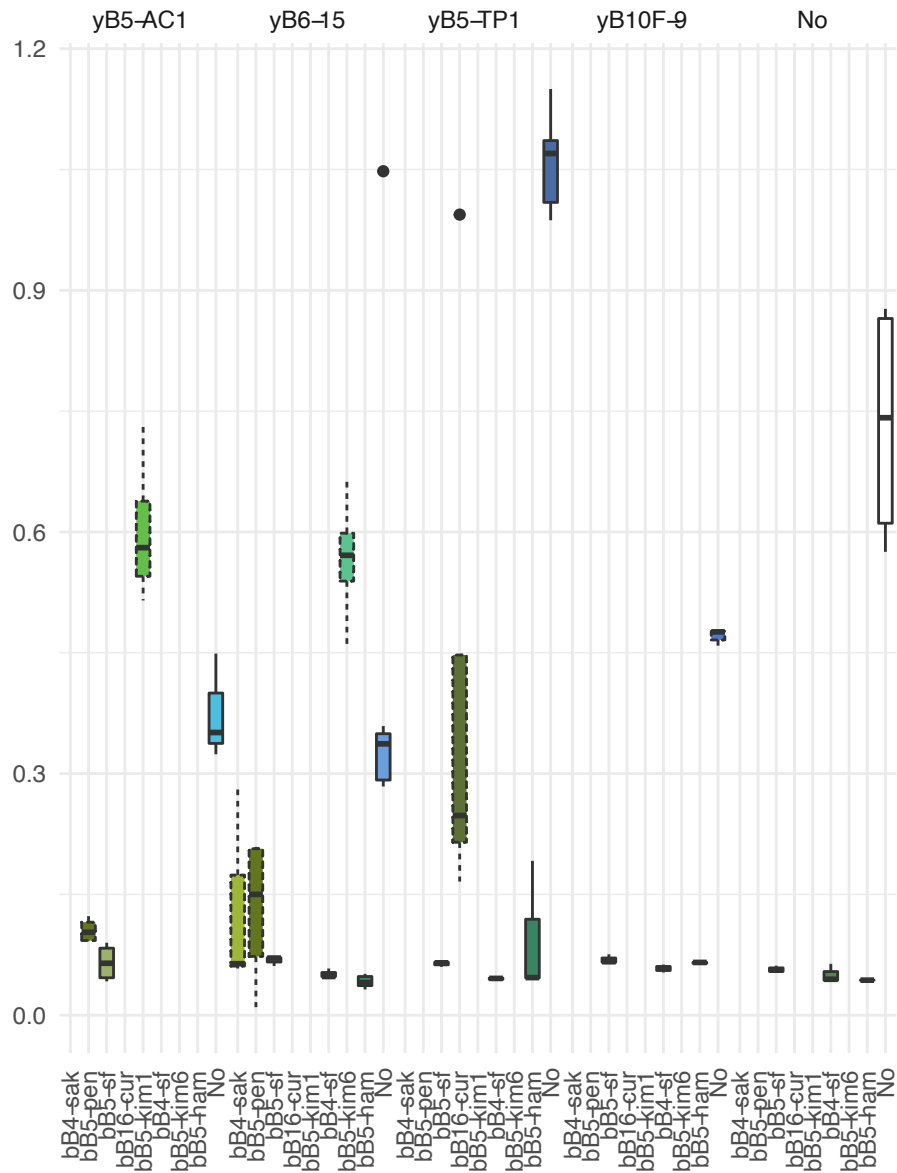

## mannitol

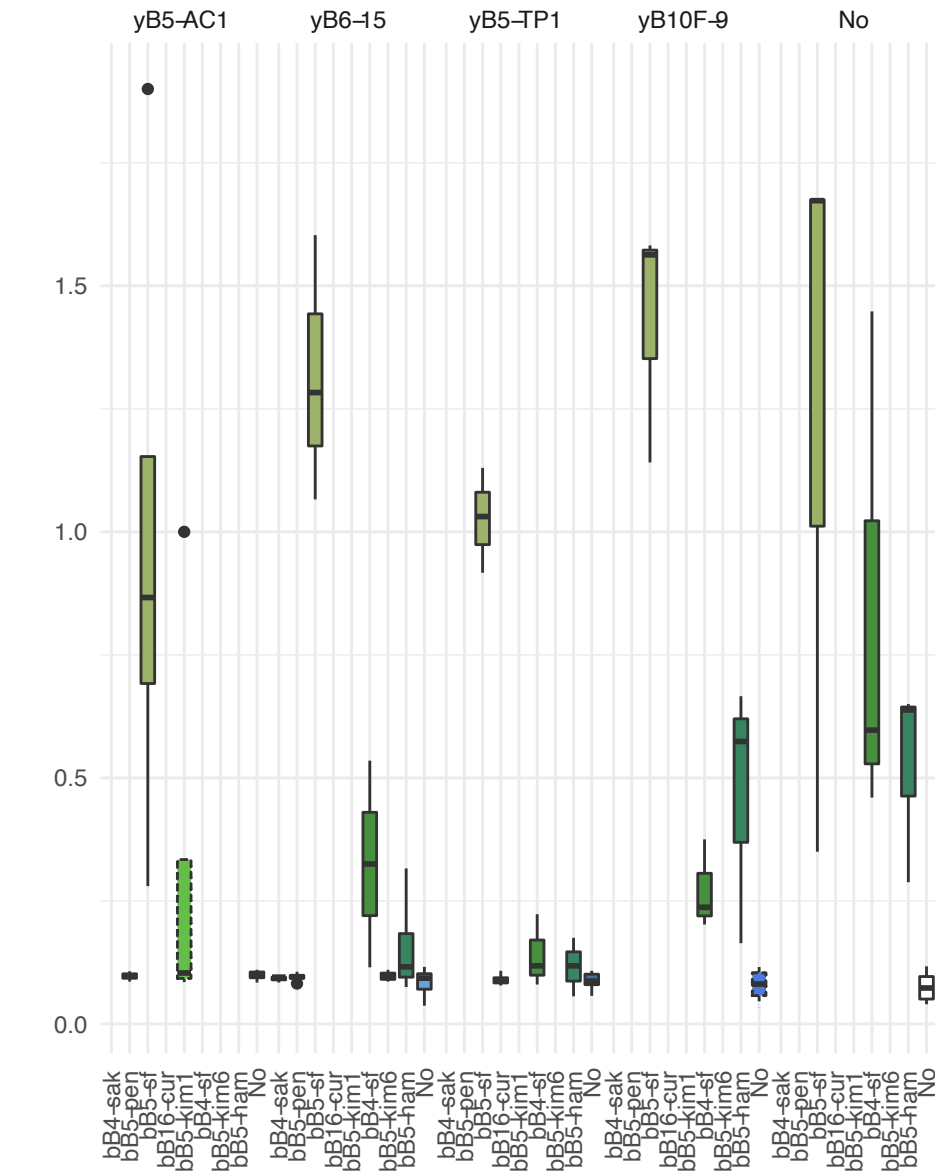

pH

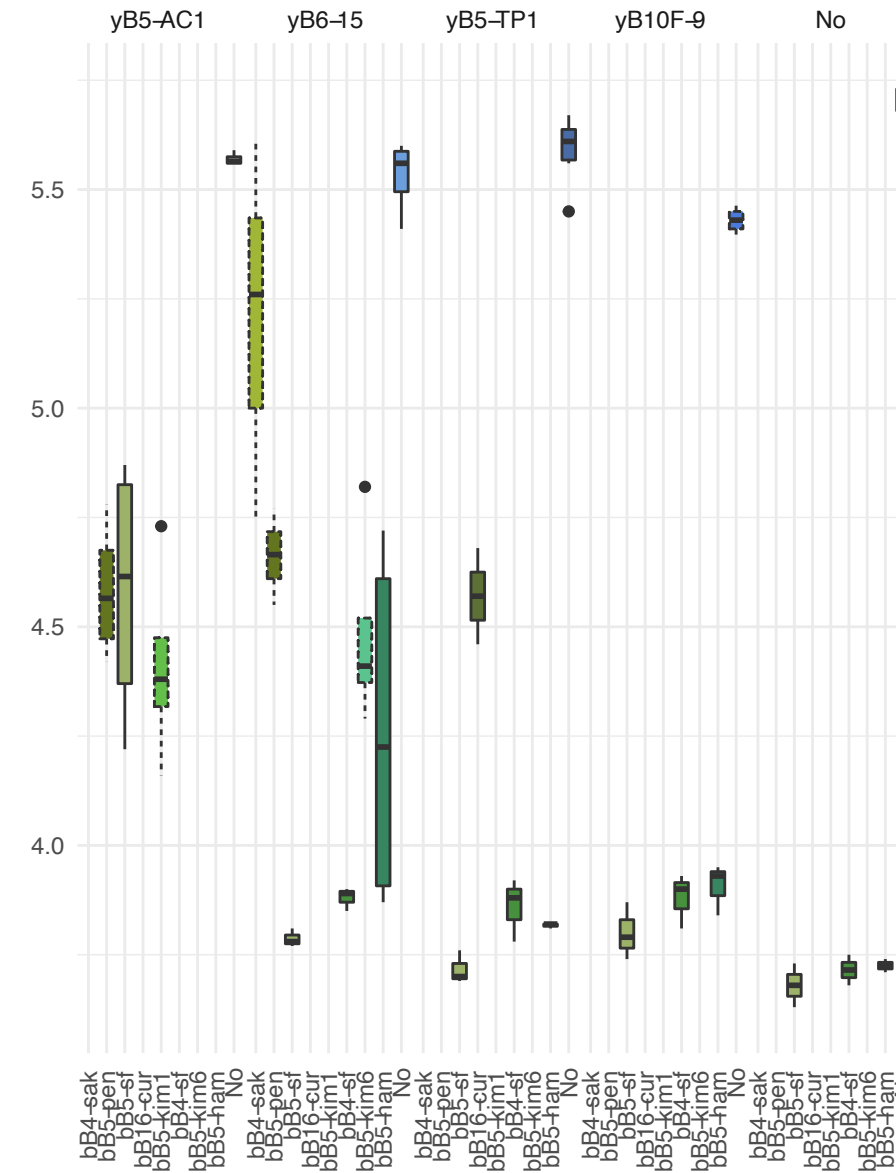

Supplement: Supplementary file 1 [file microorganisms-08-00240-s001.zip › microorganisms-681294-supplementary-final/figureS5.pdf]
